# Multi-Dimensional Prioritization of Dental Caries Candidate Genes and Its Enriched Dense Network Modules

**DOI:** 10.1371/journal.pone.0076666

**Published:** 2013-10-11

**Authors:** Quan Wang, Peilin Jia, Karen T. Cuenco, Eleanor Feingold, Mary L. Marazita, Lily Wang, Zhongming Zhao

**Affiliations:** 1 Department of Biomedical Informatics, Vanderbilt University School of Medicine, Nashville, Tennessee, United States of America; 2 Department of Human Genetics, University of Pittsburgh, Pittsburgh, Pennsylvania, United States of America; 3 Center for Craniofacial & Dental Genetics, Department of Oral Biology, School of Dental Medicine, University of Pittsburgh, Pittsburgh, Pennsylvania, United States of America; 4 Department of Biostatistics, University of Pittsburgh, Pittsburgh, Pennsylvania, United States of America; 5 Department of Biostatistics, Vanderbilt University School of Medicine, Nashville, Tennessee, United States of America; 6 Center for Quantitative Sciences, Vanderbilt University Medical Center, Nashville, Tennessee, United States of America; 7 Department of Psychiatry, Vanderbilt University School of Medicine, Nashville, Tennessee, United States of America; 8 Department of Cancer Biology, Vanderbilt University School of Medicine, Nashville, Tennessee, United States of America; Yale University, United States of America

## Abstract

A number of genetic studies have suggested numerous susceptibility genes for dental caries over the past decade with few definite conclusions. The rapid accumulation of relevant information, along with the complex architecture of the disease, provides a challenging but also unique opportunity to review and integrate the heterogeneous data for follow-up validation and exploration. In this study, we collected and curated candidate genes from four major categories: association studies, linkage scans, gene expression analyses, and literature mining. Candidate genes were prioritized according to the magnitude of evidence related to dental caries. We then searched for dense modules enriched with the prioritized candidate genes through their protein-protein interactions (PPIs). We identified 23 modules comprising of 53 genes. Functional analyses of these 53 genes revealed three major clusters: cytokine network relevant genes, matrix metalloproteinases (MMPs) family, and transforming growth factor-beta (TGF-β) family, all of which have been previously implicated to play important roles in tooth development and carious lesions. Through our extensive data collection and an integrative application of gene prioritization and PPI network analyses, we built a dental caries-specific sub-network for the first time. Our study provided insights into the molecular mechanisms underlying dental caries. The framework we proposed in this work can be applied to other complex diseases.

## Introduction

Dental caries (also known as tooth decay) is a chronic disease with high prevalence worldwide. The occurrence and progression of caries are known to be influenced by numerous environmental factors, including microbial flora, salivary flow and composition, and fluoride exposure, among others. Despite the environmental contributions, the impact of genetic factors has been recognized for a long time [1], with heritability being estimated to be between 40% and 60% [2], [3], [4]. Over the past decade, a great number of studies have been published using a variety of experimental designs and technologies, including genetic association studies [5], [6], [7], [8], genome-wide linkage scans [9], and expression profiles [10], [11], [12], aiming to dissect the genetic architecture of dental caries. A wealth of molecular genetic data has been thus accumulated rapidly. However, results across different studies are often influenced by various factors such as experiment designs, sample sizes and ethnicities, and analysis methods. Therefore, a comprehensive investigation by integrating data and information from heterogeneous sources may broaden our knowledge of caries pathogenesis. Such concept has been implemented by some computational gene prioritization approaches whose goal is to provide a ranked list of genes with combined evidence, depicting their biological relevance to disease [13], [14], [15]. Most of those integration approaches are similar and need two input gene sets: a small list of genes for training purpose and a large list of candidate genes for ranking. Training genes are usually well-studied or verified for the disease in investigation. For candidate genes, the prioritization is based on the relationships between them and training genes upon different evidence (e.g., the co-expression level of two genes in an expression profiling study). Thus, each candidate gene will have multiple ranks due to various types of evidence. Finally, statistical models are adopted to combine multiple ranks into a global prioritization.

However, the interpretation of such prioritized candidate genes is often challenging. Although pooled information may yield additional knowledge, the prioritization result is difficult to evaluate due to the lack of a generally accepted benchmarking strategy [16], [17]. Hence, the prioritized results are necessarily followed by a systematic biological exploration. Now investigators have accepted the notion that complex diseases or traits are influenced by multiple genes and the complicated interplays or interactions between them [18], [19]. So the gene set enrichment approaches are proposed to investigate the biological roles by conceptualizing a function through a predefined pathway (e.g., a KEGG [20] pathway) or a Gene Ontology (GO) [21] term [22]. One representative example of such approaches is the Gene Set Enrichment Analysis (GSEA) family, which studies the distribution of genes from the same pathway across a list of genes ranked according to differential expression [23], genome-wide association studies (GWAS) [24], and others [25], [26]. Nevertheless, clustering genes through predefined pathways or functional annotations may lead to a poor understanding of the cellular complexity in some conditions [27]. The knowledge of current pathways or ontologies is incomplete and thus has limited us to identify a meaningful combination of genes, especially for those diseases or traits that are not well studied (e.g., dental caries). The human interactome, that is, the whole protein-protein interactions (PPIs) in humans, include the functional relationships among gene products. Searching genes and their interactions in the PPI network is more flexible and dynamic, allowing us to find enriched functional interactions of candidate genes beyond the canonical pathway annotations. One rationale of the PPI approaches is that proteins often tend to interact with each other if they involve in the same physiopathogenic processes [28], [29]. So far, PPI-based analyses have been applied in numerous genetic studies [30], [31], [32].

In this study, we aimed to rank candidate genes from heterogeneous data sources for dental caries and then investigate their functional interactions through module search of the ranked candidates that were mapped onto the human PPI network. We collected all currently available candidate genes from multiple sources, including genetic association studies, genome-wide linkage scans, gene expression studies, and literature mining. These reported genes had been weighted in the original studies. However, the evidence employed to weight genes can hardly be treated consistently due to the inherent distinctiveness of experiment designs or platforms, analytical strategies, sample sizes, among others. To mitigate the inconsistency, we adopted a gene prioritization method, ENDEAVOUR [13], to obtain a global ranking of candidate genes. This resulted in a full list of prioritized genes. To focus on a subset of genes with plausible biological functions, we incorporated the PPI data to search for the dense modules enriched with the prioritized genes by applying a sub-network searching tool, dmGWAS [33]. This analysis resulted in 23 dense modules comprising of 53 genes. Three major gene clusters were observed among those 53 genes: cytokine network relevant genes, matrix metalloproteinases (MMPs) family, and transforming growth factor-beta (TGF-β) family, all of which have been previously implicated to play important roles in tooth development and carious lesions. To our knowledge, this is the first study to prioritize candidate genes and then to interpret the prioritized results through the perspective of PPI for dental caries. Our findings provided biological insights into the potential molecular mechanisms underlying dental caries, which helps to improve our understanding of the disease beyond the single gene strategies.

## Materials and Methods

**Figure 1:**
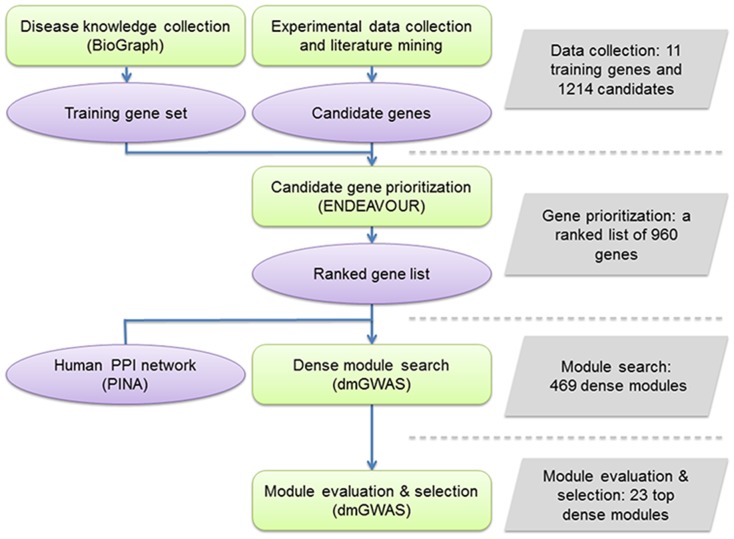
The workflow of this study. First, the biological knowledgebase BioGraph [34] was explored to identify training gene set, and candidate genes were collected from previous studies and publications. We obtained 11 training genes and 1214 candidate genes in this data collection step. Second, a computational method ENDEAVOUR [13] was utilized to prioritize the candidate genes. In this step, a ranked list of 960 candidate genes that could be recognized by ENDEAVOUR was generated. Third, dmGWAS [33] was employed to search for the dense modules upon human protein-protein interaction (PPI) network collected by Protein Interaction Network Analysis (PINA) platform [53]. This resulted in 469 dense modules. Finally, the 469 modules were evaluated and the top 23 ones were selected as promising modules.

We developed a computational framework to prioritize dental caries genes from multiple sources and then search for enriched modules of the highly ranked genes followed by module evaluation. Figure 1 illustrates the workflow. It consists of four steps: data collection, gene prioritization, module search, and module evaluation. For data collection, we prepared two sets of genes: (1) training genes, which were generated by exploring a comprehensive biomedical knowledge database BioGraph [34], and (2) candidate genes, which were collected and curated from previous genetic studies and literatures. Then, we applied a gene prioritization method, ENDEAVOUR [13], to rank the candidate genes. Next, we employed dmGWAS [33] to search for modules by mapping the prioritized genes onto the human PPI networks. Finally, we evaluated the generated modules and selected promising ones for further investigation or discussion.

### Candidate Gene Collection

Candidate genes were collected from multiple sources, including association studies, linkage scans, gene expression, and literature mining. Although the first genetic research for dental caries could be traced back to 1930s [35], there are not as many high-throughput experiments have been performed for dental caries as for other complex diseases (e.g., cancer, diabetes). This is largely due to the difficulty in sample preparation [10], [12]. Therefore, in addition to the genes collected from genetic studies (association, linkage scans, and gene expression), we also conducted literature mining to obtain more caries related candidate genes. To address the great variety of data, we explicitly grouped candidate genes into four categories: ‘association’, ‘linkage’, ‘expression’, and ‘literature’. The collection procedure for each category is described below.

#### Association studies

Candidate genes reported in association studies were collected via searching the published articles. One recent review [35] offered an overview of genetic influence on dental caries. It reviewed eight individual association studies (as of 2010) that showed evidence for genetic influence to tooth decay susceptibility. We manually checked these publications and extracted 12 caries related genes. In addition, we searched NCBI PubMed database for the genetic association studies published after 2010 or those that were not included in [35]. As of January 31, 2013, we found a total of 14 association studies for dental caries [4], [5], [6], [7], [8], [36], [37], [38], [39], [40], [41], [42], [43], [44], including three genome-wide association (GWA) studies [8], [43], [44]. Shaffer and colleagues [8] conducted the first GWA study on caries and suggested several loci (*ACTN2*, *MTR*, *EDARADD*, *MPPED2, LPO, EPHA7,* and *ZMPSTE24*) with plausible biological roles in the susceptibility to childhood caries. The other two GWA studies [43], [44] focused on caries in the permanent dentition. The association analyses were performed on novel dental caries patterns [43] and independent cohorts [44], which resulted in 12 and 6 genes that were implicated to harbor genetic association signals respectively. For non-GWA studies, we manually scrutinized the 11 remaining papers and extracted 18 association-based candidate genes. Putting together, we obtained 55 non-redundant genes from association studies (Table 1). It is worth noting that the samples recruited in these published association studies encompass diverse ages and populations. For instance, the samples recruited in the first GWAS [8] had age range between 3 and 12 years old, while the other one [43] focused on participants with ages between 18 and 75 years. The association between *AMELX* polymorphisms and caries were found in Korean children [7], while another study [38] suggested HLA class II allele as a susceptibility locus in Brazilians. Nevertheless, this is the largest collection of association genes for dental caries.

**Table 1:** Dental caries candidate genes in four categories.

Category	# candidate genes	Reference
Association	12	[35]
	8	[8]
	12	[43]
	6	[44]
	20	[4]–[7], [36]–[42]
Linkage	349	[9]
Expression	13	[10]
	324	[11]
	8	[12]
Literature	570	–
Total^a^	1214	–

a The total number is smaller than the sum of the four categories due to redundancy.

Linkage scans. So far, there has been only one linkage scan for dental caries [9]. In that study, Vieira and colleagues scanned 46 families with similar cultural and behavioral habits. The loci whose logarithmic odds (LOD) scores were greater than 2 or p-values were less than 0.0009 were considered as risk susceptibility. According to these criteria, the original study reported five susceptibility loci (5q13.3, 14q11.2, Xq27.1, 13q31.1, and 14q24.3). We mapped these five loci to the human genome (hg18) by their corresponding physical locations, and then extracted the genes within these genomic regions. This resulted in a total of 349 linkage-based candidate genes (Table 1).

#### Gene expression data

There are only a few high-throughput characterization studies of gene expression profiling under carious lesion due to the difficulties in collecting the sufficient amount of dental tissues [10], [12]. We searched the NCBI PubMed database and collected two published high-throughput gene expression studies using microarray techniques [11], [12] and one small scale gene expression study for caries [10]. In [11], gene expression of 12 healthy and 11 carious teeth was screened using human Affymetrix HG_U133A oligonucleotide arrays (readers are referred to the original publication for more details). In [12], the investigated samples were 42 sound and 62 carious molars, and the platform was Atlas Glass Human 1.0 microarray. We manually checked the differentially expressed genes provided in the original papers and selected only those with at least 2-fold change. Correspondingly, we obtained 324 and 8 differentially expressed genes from [11] and [12], respectively. In the third gene expression study for carious teeth [10], histological findings indicated DSPP and NES were promising genes in pulpal tissues. By using semi-quantitative reverse transcriptase polymerase chain reaction (sq-RT-PCR), the authors verified the gene expression of DSPP, NES, and related genes in healthy and carious teeth [10]. We obtained 13 differentially expressed genes from that study. Collectively, we gathered 344 non-redundant differentially expressed genes from three independent studies (Table 1).

#### Literature mining

**Figure 2:**
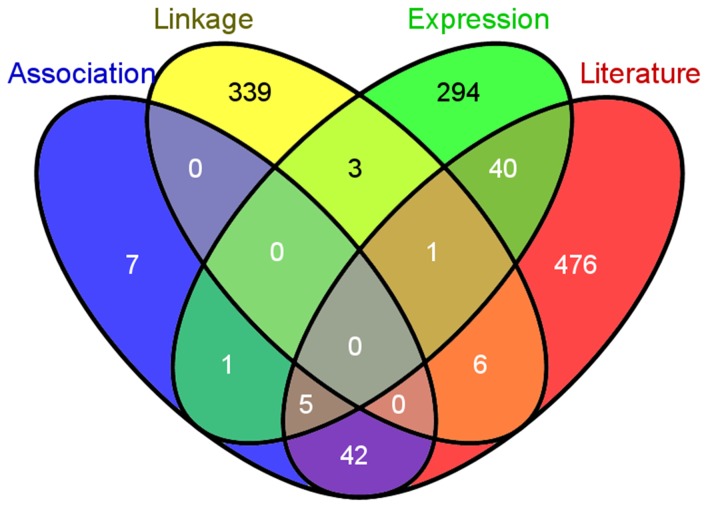
Overlap of candidate genes between four categories.

Literature mining was performed by searching NCBI PubMed for the co-occurrence of two entries in title/abstract: a gene name and a caries-related item. All human coding genes (hg18) were included for searching. Three keywords (‘dental caries’, ‘tooth decay’, and ‘teeth decay’) were selected by experts in dental caries research and used for the literature mining. If a gene and any of the three keywords co-occurred in the same publication, a hit would be assigned to the gene. For example, gene CXCL10 and keyword ‘dental caries’ co-occurred in 5 publications; thus, 5 hits were assigned to CXCL10. After a systematic search of the combination of all genes and keywords in PubMed, we manually examined the results by removing genes with special symbol names (e.g., GRASP, LARGE, MAX). A total of 570 genes were collected with at least one hit. Note that genes identified by literature mining may overlap with previously collected candidate genes, but number of hits has the weight of genes in this category (Figure 2).

### Training Gene Set

The reliability of training genes is critical to the gene prioritization [16]. However, few of the reported caries genes have been rigorously replicated or confirmed to date [43]. Instead of using such gold standard genes, we explored a comprehensive database, BioGraph [34], to identify genes for training purpose. BioGraph is a data mining platform for the discovery of biomedical knowledge. It integrates 21 publicly available curated databases encompassing multiple relationships between heterogeneous biological concepts such as genes, proteins, diseases, pathways, and ontology terms. Based on these integrated databases, BioGraph generates an interaction map linking different biological concepts. By fixing a specific biological concept (e.g., a disease) as a potential target, it provides an online resource (http://www.biograph.be/) to extract the most significant links between other biological concepts and this target. Setting ‘dental caries’ as our disease target, a list of related concepts could be obtained from BioGraph, such as ‘gene’, ‘compound’, ‘pathway’, among others. We only focused on ‘gene’ while excluding all the other concepts. Through manually scrutinizing the top 100 related genes from BioGraph, we found 11 genes (*IL6*, *IL8*, *AMELX*, *MMP2*, *TAS1R2*, *INS*, *DSPP*, *TNF*, *TRPV1*, *HLA-B*, and *LTF*) had literature support. The literature evidence stretched over multiple experiment types. Specifically, six genes (*AMELX, MMP2, TAS1R2, DSPP, HLA-B,* and *LTF*) were supported by genetic association studies [4], [6], [7], [36], [38], [39], [40], [41], [45], [46], [47]; four genes (*IL6, IL8, TNF,* and *TRPV1*) were supported by expression analyses [48], [49], [50], [51]; and gene *INS* was supported by a contrast trial [52]. These 11 genes were used as training genes in our downstream analysis.

### Candidate Gene Prioritization

We utilized ENDEAVOUR [13] to carry out gene prioritization. ENDEAVOUR is a prioritization method integrating the prior disease knowledge and fused genomic data to rank candidate genes for the disease. Based on the gene similarity originated from multiple heterogeneous data sources, ENDEAVOUR adopts statistical models to investigate the matching qualities between candidate genes and training genes. The order statistics based p-values, which are transformed from the matching qualities, are then used to generate a global gene ranking. More details of the algorithm can be found in the original publications [13].

### Dense Module Search

Upon the prioritized genes, we employed dmGWAS [33] to search for dense modules by incorporating human PPI data. The human PPI data was downloaded from Protein Interaction Network Analysis (PINA) platform [53]. PINA integrated PPI data from six manually curated databases (HPRD, IntAct, DIP, MINT, BioGRID and MIPS/MPact). To ensure the reliability, we only retained the interactions with experimental evidence. As of January 31, 2013, approximately 13,000 nodes and 101,000 interactions with experimental evidence were included in PINA. We utilized the data to build a whole human PPI network, upon which dmGWAS could search for dense modules. dmGWAS was specifically designed for searching combined association signals from a GWAS dataset [33] or multiple GWAS datasets [30]. Each gene was assigned a p-value from the GWAS signals. From a seed module (at first a single gene in the PPI network), dmGWAS expanded the module via investigating its neighborhood nodes. If the p-value of a neighborhood gene was significant enough (a dynamic threshold was designed to determine the significance based on its transformed score), it would be added into the module. This step was repeated until no more nodes could be added. dmGWAS reports the constructed sub-network as a final module. Readers may find more details in the original publication [33]. In this study we applied the underlying algorithm of dmGWAS to select highly ranked and interconnected candidate genes and built a disease-specific sub-network. Specifically, we substituted the GWAS p-values of candidate genes with the order statistics based p-values from ENDEAVOUR’s results.

### Dense Module Evaluation

In addition to search for the dense modules, dmGWAS provides a procedure to evaluate them. Briefly, a module score 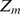 is computed as 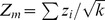, where 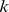 is the number of genes in the module. For each gene 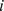, 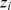 is transformed from its p-value 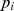 according to 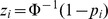, where 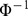 is the inverse distribution function of the standard normal distribution. Under this scoring system, a larger 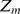 means that the corresponding module holds a higher proportion of low p-value genes. However, the connection between ENDEAVOUR’s prioritization and network topology is not reflected in the module score 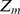. Considering this, 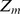 is then calibrated to determine whether it is higher than expected relative to a random set of genes selected from the PPI network. Specifically, for a module with 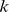 genes, a background distribution of module scores 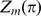 is generated by computing 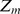 through randomly choosing the same number of genes from the whole network 100,000 times. Accordingly, 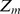 is normalized as 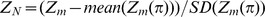, where SD is standard deviation. Under this correction, 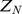 reflects information resided in both gene ranking and network topology and, thus, can be used to select the modules with enriched signals.

## Results

### Comparison of Candidate Genes in Four Data Categories

In order to collect a list of candidate genes as comprehensive as possible, we performed an extensive and systematic search of publications and carefully curated the results from them. Table 1 summarizes the collected data, representing the most comprehensive collection and curation of candidate genes for dental caries to date. The number of genes varied greatly among categories, reflecting the scale of the studies and resolution of the data generated by different technologies. After removing the redundancy, we obtained a union of 1214 candidate genes (Table 1 and Figure 1). Among them, no genes belonged to all four categories; 6 genes belonged to three categories; 92 genes belonged to two categories; and the remaining 1116 genes were found only in one signal category (Figure 2). As shown in Figure 2, the genes from linkage scan displayed fewer overlap with all the other three categories (‘association’, ‘expression’, and ‘literature’). This might be because only one study was conducted for linkage analysis of dental caries (Table 1).

### Candidate Gene Prioritization

ENDEAVOUR provides a freely accessible web service interface (http://homes.esat.kuleuven.be/~bioiuser/endeavour/index.php) for users. The 11 training genes and 1214 candidate genes in our data collection were used to perform gene prioritization by ENDEAVOUR. Specifically in this analysis, for the prior knowledge used to establish the link between training and candidate genes, we excluded four PPI interaction databases (HPRD, IntAct, MINT, BioGRID) because they were included in the PINA PPI data upon which we would search for dense modules. Order statistics were employed to combine the rankings based on each line of evidence, and then integrated p-values were computed from order statistics. Note that a smaller p-value indicates a higher ranking. In this procedure, we obtained a prioritized list of 960 genes whose gene symbols could be recognized by ENDEAVOUR (Figure 1 and Table S1). Not surprisingly, most of the training genes had the higher rank than other candidate genes. Ten of the 11 training genes were among the top 20 in the ranked gene list. The remaining training gene, *TAS1R2*, ranked the 47^th^ (Table S1). We examined the correlation between gene ranking and the number of its source categories. As expected, the top ranked genes tended to have more source categories (Table 2).

**Table 2:** The top ranked genes have a higher probability of belonging to multiple categories.

	# source categories			p-value^a^
	1	2	3	
Full prioritized list	862	92	6	–
Top 50 genes^b^	30	18	2	1.79×10^−7^
Top 100 genes	67	29	4	1.08×10^−8^
Top 200 genes	154	40	6	4.37×10^−6^

a p-values computed by Fisher’s exact test.

b The top 50 genes in the prioritized candidate gene list by ENDEAVOUR [13].

### Dense Module Search and Evaluation

**Figure 3:**
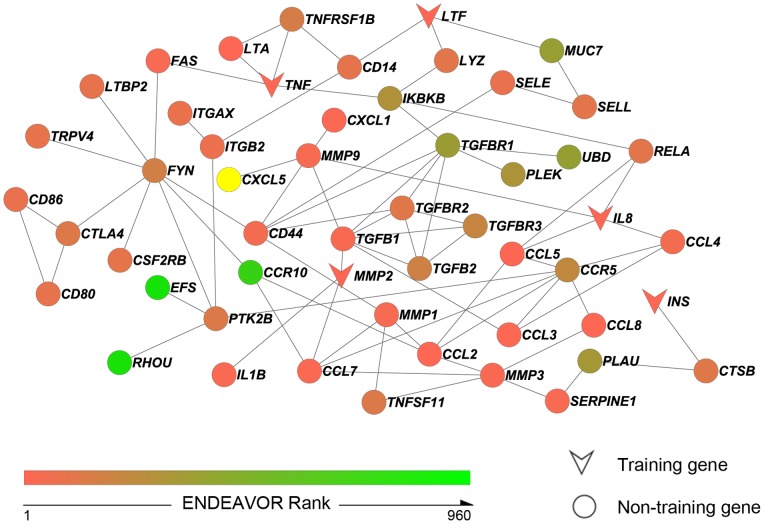
The sub-network containing 53 DCgenes from the selected 23 modules (top 5% of all modules generated by dmGWAS). Three gene clusters with plausible functions were included: cytokine network relevant genes (*CCL2, CCL5, CCL8, CCL3, CXCL1, CXCL5, CCL7, CCL4, CCR5,* and *CCR10*), matrix metalloproteinases (MMPs) family genes (*MMP2, MMP3, MMP1,* and *MMP9*), and transforming growth factor-beta (TGF-β) family genes (*TGFB1, TGFBR2, TGFB2, TGFBR3,* and *TGFBR1*), all of which have been previously implicated to play important roles in tooth development and carious lesions.

We utilized dmGWAS to search for dense modules enriched with highly ranked genes. With the default parameters, dmGWAS generated a total of 469 modules (Figure 1). On average, the module size (measured by gene number) was 10.31±2.28 (mean ± SD). As described in the Materials and Methods, the normalized score 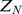 was used to assign significance of the 469 modules. To select biologically meaningful modules, one straightforward way is to transform 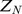 back to p-values by 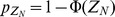. However, all the 469 modules would be nominally significant (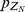 within a range of 1.23×10^−17^–1.60×10^−5^) if the threshold were set as 0.05. In addition, it is not appropriate to perform multiple testing correction directly because dmGWAS introduces extensive overlap between modules. Thus, similar to the original study [33], we selected the top 5% modules for downstream analysis. Although this selection is somewhat arbitrary, we considered it appropriate to focus on moderate interesting genes while not including too many unrelated modules. Applying this criterion resulted in 23 modules (Figure 1). Those modules comprised of 53 non-redundant genes, including five training genes (*IL8, MMP2, LTF, TNF,* and *INS*). We mapped the 53 genes back to the whole PPI network and extracted the edges between them to form a sub-network, which was visualized by network software Cytoscape [54] (Figure 3). The detailed gene information was provided in Table 3. We termed these 53 genes dental caries genes (DCgenes) hereafter.

**Table 3:** The 53 DCgenes residing in the top 23 dense modules.

Gene symbol	Training gene	Source	ENDEAVOUR rank	Degree^a^
*IL8*	Yes	Expression, Literature	1	4
*MMP2*	Yes	Association, Literature	3	3
*LTF*	Yes	Association, Literature	4	3
*TNF*	Yes	Literature	5	4
*INS*	Yes	Literature	6	1
*CCL2*	No	Expression, Literature	7	5
*CCL5*	No	Expression, Literature	11	4
*CCL8*	No	Expression, Literature	12	2
*CCL3*	No	Expression, Literature	13	3
*LTA*	No	Literature	14	2
*CXCL1*	No	Expression, Literature	15	1
*CXCL5*	No	Expression	16	1
*CCL7*	No	Literature	19	5
*IL1B*	No	Expression	20	1
*MMP3*	No	Association, Literature	25	5
*FAS*	No	Literature	26	2
*SERPINE1*	No	Literature	27	2
*TGFB1*	No	Expression, Literature	30	7
*MMP1*	No	Expression	33	4
*MMP9*	No	Expression, Literature	36	5
*CD44*	No	Expression, Literature	42	6
*CCL4*	No	Literature	45	3
*ITGB2*	No	Expression	71	3
*CD86*	No	Literature	80	2
*SELE*	No	Expression	82	2
*ITGAX*	No	Literature	85	1
*LTBP2*	No	Linkage	89	1
*CD80*	No	Literature	93	2
*TRPV4*	No	Literature	95	1
*CD14*	No	Association, Expression, Literature	97	3
*CSF2RB*	No	Expression	100	1
*RELA*	No	Literature	108	3
*SELL*	No	Literature	111	2
*LYZ*	No	Expression	116	2
*TGFBR2*	No	Expression	122	5
*CTSB*	No	Expression	126	2
*CTLA4*	No	Literature	127	3
*TNFSF11*	No	Literature	132	2
*PTK2B*	No	Association, Literature	136	5
*TNFRSF1B*	No	Expression	155	3
*FYN*	No	Literature	176	8
*TGFB2*	No	Expression	189	4
*TGFBR3*	No	Expression	219	3
*CCR5*	No	Literature	238	7
*IKBKB*	No	Literature	304	4
*PLEK*	No	Expression	319	1
*PLAU*	No	Expression	340	2
*TGFBR1*	No	Expression	377	7
*MUC7*	No	Literature	394	2
*UBD*	No	Expression	398	1
*CCR10*	No	Literature	756	3
*RHOU*	No	Association, Literature	871	1
*EFS*	No	Linkage, Literature	880	1

a The degree of a node is the number of its neighbors in the sub-network.

As shown in Table 3 and Figure 3, one training gene (*IL8*) ranked the highest, followed by four other training genes (*MMP2, LTF, TNF,* and *INS*). However, not all the DCgenes had a higher gene ranking. Specifically, 31 genes ranked above the 100^th^ while 22 genes were below the 100^th^. Of note, dmGWAS identified three genes (*CCR10, RHOU,* and *EFS*) that ranked below the 700^th^. The genes with lower ranking were selected by dmGWAS because they had protein interactions with the training or highly ranked genes, an advantage in the network-assisted approach. In addition, we investigated the degrees of the DCgenes. Nearly half of them (25, 47.17%) had only one or two neighbors. There were 11 proteins whose degrees ≥5. Among them, FYN had the highest degree, i.e., 8. Interestingly, two C-C chemokine ligands (CCL2 and CCL7), two MMPs (MMP3 and MMP9), and three TGF-β family members (TGFB1, TGFBR1, and TGFBR2) had relatively high degrees. Besides, three other proteins (CCR5, CD44, and PTK2B) had degrees ≥5.

We further explored the functions of these DCgenes. Dental caries has been known as an inflammatory disease for a long time [55]. The oral environment contains bacteria that might stimulate the host’s inflammatory response eliciting cytokines [56], [57], [58]. Two interleukin genes (*IL8* and *IL1B*) have been shown to play important roles in cytokine secretion in saliva and odontoblast layer of human teeth [48], [59]. The C-C chemokine ligand (CCL), C-C chemokine receptor (CCR) and C-X-C chemokine ligand (CXCL) gene family have been previously investigated and reported to increase expression level of various cytokines in carious pulp and/or odontoblast [59]. Our DCgenes list covered a number of CCL, CCR and CXCL genes, including *CCL2, CCL5, CCL8, CCL3, CXCL1, CXCL5, CCL7, CCL4, CCR5,* and *CCR10*. Another interesting gene group in the merged sub-network belonged to the MMP family. The MMPs have been well acknowledged to be involved in the caries process by previous studies [5], [41], [42]. We identified four genes belonging to the MMP family: *MMP2, MMP3, MMP1,* and *MMP9*. Additionally, five TGF-β family members (*TGFB1, TGFBR2, TGFB2, TGFBR3,* and *TGFBR1*) comprised another interesting cluster, which has been extensively studied previously [10], [60], [61], [62].

## Discussion

A wealth of genetic data for dental caries accumulated rapidly over the past several years. In this study, we aimed to uncover the molecular mechanisms and polygenic interactions underlying this prevalent disease through systematic data integration and analyses. We collected data from four major sources, including genetic association studies, linkage scans, gene expression analyses, and literature mining. Then, the relevant genes and the interplays between them were searched based on a prioritized list of the collected candidate genes. Our study provided not only a manually curated gene database for dental caries, but also a list of promising genes and their interactions that deserve further biological investigation.

There are a few existing biomedical databases that gather information of susceptibility genes by text mining or literature review for complex diseases, such as the HuGE Navigator [63] and the Genetic Association Database (GAD) [64]. However, without an extensive publication exploration or manual check, such databases typically include broad knowledge and are usually less informative or complete for each specific disease, especially for not well-studied diseases like dental caries. For instance, when we searched for dental caries information in the HuGE Navigator (May 2013 version), we found only 58 relevant genes, all of which were collected from genetic association studies. However, our manual check of these genes suggested that some of them might be included by errors. For instance, gene *MMP14* was collected because it was one of the genes investigated in an association study [41]. However, we found the association signal of *MMP14* from study [41] was not significant after our manual check of the original work. In addition, HuGE Navigator provides no information of linkage scan or gene expression analysis. In contrast, we integrated data across multiple categories and manually checked all of them.

In addition to the data collection and integration, we performed gene prioritization to assess the priorities of candidate genes. Evidence has been shown that a single risk factor, such as a susceptibility gene, may not entirely explain the dental caries development [35]. So we attempted to explore its physiopathogenic processes through searching for dense modules enriched with prioritized candidate genes. The PPI network assisted approaches have been successfully employed to investigate other complex diseases [30], [31]. Of note, the dense module search is sensitive to the reference network. In every step of module expanding, dmGWAS examines all the neighborhood nodes and recruits the nodes with the strongest signal. Thus, the reliability of the connections between nodes is crucial for module expansion. Additionally, the current knowledge of human PPI network is far from complete. To ensure the reliability of PPI data, we restricted our work on the interactions with experimental evidence while excluding interactions predicted by computational methods. By incorporating PPI network, we selected some interesting genes with comparatively low ranking. For example, two genes *PTK2B* and *RHOU* were suggested to be candidate loci previously, and they are involved in pathways that have been implicated to dental caries [44]. However, neither of them ranked high. *PTK2B* ranked 136^th^ and *RHOU* nearly hit the bottom of the prioritized gene list (Table 3). This result implies that an inference only based on gene prioritization might miss disease signals, but network-assisted approach could help to detect a set of genes whose combined roles might involve in disease development.

The list of DCgenes (Table 3) identified within top dense modules may warrant further investigation. Bacterial invasion plays a crucial role in the development of dental caries [65]. It is also well known that cytokines are important to maintain host response to microbial infection [48], [66]. Living cells of the host secrete the molecules (such as chemokines, pro-inflammatory cytokines, and anti-inflammatory cytokines) to keep a balanced oral environment for tissue repair [67]. Various cytokines have been investigated in previous studies [48], [66]. Recently, Host and colleagues [59] systematically measured cytokine gene expression levels within human teeth that were under response to caries and built fine-tuned cytokine and chemokine signaling networks. It is worth noting that the vast majority of genes measured by microarray in [59] are human inflammatory cytokines and receptors. To avoid being overwhelmed with cytokines genes, we did not include differentially expressed genes reported in [59] in data collection. Interestingly, our DCgenes list covered considerable relevant genes, including *IL8, IL1B, TNF, CCL2, CCL5, CCL8, CCL3, CXCL1, CXCL5, CCL7, CCL4, CCR5,* and *CCR10* (Table 3 and Figure 3), and thereby offered independent evidence to reinforce the link between cytokine network and dental caries. All the relevant genes listed above could be found in the networks built in [59] except *CCR10*. *CCR10* was incorporated in our merged sub-network due to molecular interactions with *CCL2* and *CCL7* (Figure 3). This result suggests that our work could provide new knowledge about cytokine network induced by caries.

Compared to other plausible genetic risk factors of dental caries, MMPs have raised much more attention for a long time. They have been well documented to play various roles in the organization of enamel and dentine organic matrix, suggesting their contributions to the control and progression of carious lesions [68], [69], [70], [71], [72]. For instance, MMP2 was demonstrated to cleave amelogenin, the major structural protein of human enamel, into several fragments of differing molecular masses and therefore, play a curial role during tooth development [72]. Another study [73] revealed different expression patterns of *MMP2* between caries and sound dentine. In the light of importance of MMPs, a few association studies recently have been conducted in order to investigate the impact of genetic variants in MMPs [5], [41], [42]. For instance, the allele frequencies of *MMP2* and *MMP13* were found to be different between caries-affected and caries-free samples. The plausible biological functions of MMPs for dental caries were also reflected in this study. Our DCgenes list gleaned a cluster of MMP genes, including *MMP2, MMP3, MMP1,* and *MMP9* (Table 3 and Figure 3).

Another interesting gene group we identified is the TGF-β family, which has been shown being involved in cellular signaling during tooth development and repair for a long time [61], [62], [74]. Previous studies implicated TGFB1, together with MMPs, participate in organization of dentin organic matrix remodeling [75], [76]. Two important cooperators are MMP2 and MMP9 [76], both of which were connected with TGFB1 in our merged sub-network (Figure 3). In addition, differential expression patterns of TGF-β isoforms and receptors were detected in odontoblasts and pulpal cells between human healthy and carious teeth by independent studies [10], [60], [61], suggesting their participation in tissue response to injury. The roles of the TGF-β family were also supported by our network analysis. In Table 3, we identified two TGF-β isoforms (*TGFB1* and *TGFB2*) and three TGF-β receptors (*TGFBR1, TGFBR2,* and *TGFBR3*), all of which closely interacted with each other in the merged PPI network (Figure 3).

One limitation in this study is the choice of training genes. Although a good number of susceptibility genes have been reported for dental caries, few of them have been rigorously replicated or confirmed [43]. Our selection of training genes through BioGraph was somewhat subjective, which may influence the prioritization of candidate genes. Nonetheless, the 11 training genes were manually scrutinized and supported by multiple lines of evidence. For example, one training gene, *MMP2*, has been previously implicated in cariogenesis by genetic association study [41], expression analysis [73], and immunohistochemical experiment [72]. Compared to other popular biological knowledgebases such as the HuGE Navigator, which only collects genetic association information, our training genes encompassed broader prior knowledge.

To evaluate the robustness of our findings, we randomly selected a set of candidates from the 1214 candidate genes and analyzed them with 11 training genes following the same pipeline. We repeated this procedure a few times and found the main gene clusters resided in top modules were still the cytokine genes, MMPs family, and TGF-β family, supporting our previous findings (data not shown). The approach we proposed in this work can be expanded by integration of data from other sources. For example, animal models have been frequently used to study the candidate genes’ function and their potential molecular mechanisms involved in human complex disease. Gene expression experiments had been performed in mouse caries model [77], and such data might be utilized in a cross-species data integration model [78]. Alternatively, integrative data analysis utilizing regulatory information, such as expression quantitative trait loci (eQTL) [79], methylation quantitative trait loci (mQTL) [80], microRNA and/or transcription factor regulatory network [81], has been demonstrated effective in many complex diseases.

To our knowledge, this is the first study that comprehensively collected and curated evidence-based candidate genes for dental caries. Through an integrative application of gene prioritization and PPI network analysis, we identified 53 potential susceptibility genes and their PPI interactions for this disease. Our results confirmed and expanded current knowledge in dental caries genetics, e.g., the interactions between MMPs and TGF-β family. Thus, this study provided additional biological insights and better understanding of the underlying pathological processes in dental caries. The strategy on integrating gene prioritization and PPI network analysis that we proposed in this study can be applied to other complex diseases.

## Supporting Information

Table S1 **This table provides the ENDEAVOUR ranking of 960 candidate genes and the source categories of each gene.**
(XLSX)
